# Synchronous triple primary malignant tumours in the bladder, prostate, and lung harbouring TP53 and MEK1 mutations accompanied with severe cardiovascular diseases: A case report

**DOI:** 10.1515/med-2022-0616

**Published:** 2022-12-14

**Authors:** Zhi-Ke Li, Qiang Zhao, Ning-Fu Li, Jing Wen, Bang-Xian Tan, Dai-Yuan Ma, Guo-Bo Du

**Affiliations:** Department of Oncology, The First Affiliated Hospital of North Sichuan Medical College, Nanchong 637000, Sichuan, China; School of Medical Imaging, North Sichuan Medical College, Nanchong, China; Department of Oncology, The First Affiliated Hospital of North Sichuan Medical College, No. 1 Maoyuan South Road, Shunqing District, Nanchong 637000, Sichuan, China

**Keywords:** multiple primary malignancies, non-small cell lung cancer, severe cardiovascular disease, TP53 mutations, PD-1

## Abstract

Although the incidence of multiple primary malignancies (MPMs) is increasing, synchronous triple primary malignant tumours with prostate, bladder and lung is rarely reported. Gene mutation is thought to be a reason for MPMs, and severe cardiovascular diseases may interrupt the cancer treatment. Here we reported a 64-year-old male patient with synchronous triple primary malignant tumours of the bladder urothelial carcinoma, prostate adenocarcinoma, and non-small cell lung cancer (NSCLC) with mutations in TP53 and MEK1, all the three malignancies were diagnosed within 10 days. Although being interrupted by severe cardiovascular diseases (including myocardial infarction, venous thrombosis, and aneurism of the aortic root), he was successfully treated with radical cystoprostatectomy, chemotherapy plus pembrolizumab (a PD-1 antibody), and radiotherapy of the lung lesion, followed by maintenance monotherapy of pembrolizumab, overall survival was more than 26 months. In conclusion, a patient of synchronous triple primary malignant tumours with prostate, bladder, and lung harbouring TP53 and MEK1 mutations accompanied with severe cardiovascular diseases was treated successfully, which may suggest that comprehensive treatment, especially radical treatment such as operation and radiation, is very important for MPMs.

## 1 Introduction

Multiple primary malignancies (MPMs) are the malignancies that develop in the same patient [1], the incidence of MPMs is increasing recently [2]. The reported incidence of a second primary tumour is about 3–5%, and a third one is 0.5% [3], but as accompanied with benign ones [4], the incidence of MPMs maybe more rare. Some risk factors may be associated with MPMs, including gene, hormonal factors, immune deficiency, and so on [5,6]. If the second tumour occurs in 6 months, it is defined as synchronous, while over 6 months is metachronous [7]. The most reported tumours about MPMs are from lung, breast, colorectal, prostate, gastric, and so on [8,9,10,11]. MPMs of the prostate and bladder have been reported [12], a part of the bladder cancer patients who underwent radical surgery have been diagnosed with prostate cancer thereafter [13,14]. But gene mutation is rarely reported, and a lung cancer with these two is rare [15,16], as well as synchronous triple primary malignancies with severe cardiovascular diseases.

Gene mutations are more likely to occur in multiple primary tumours. MEK1 is a typical downstream effector of activating mutant KRAS in the MAPK signalling pathway [17]. Active MAPK pathway has a key role in the expression of PD-L1 in lung adenocarcinoma [18]. It has been shown that MEK inhibition can enhance the effect of immunotherapy against PD-1/PD-L1 [19]. Therefore, we may hypothesize that MEK1 could be responsible for a better prognosis of the patient being treated with immunotherapy.

To our best knowledge, this is the first report of synchronous triple primary malignancies of the prostate adenocarcinoma, bladder urothelial carcinoma, and non-small cell lung cancer (NSCLC) with mutations in TP53 and MEK1, accompanied with severe cardiovascular diseases. The patient successfully treated with radical cystoprostatectomy, chemotherapy plus pembrolizumab (a PD-1 antibody), and radiotherapy of the lung cancer, and maintenance therapy of pembrolizumab monotherapy was still ongoing.

## 2 Case presentation

**Figure 1:**
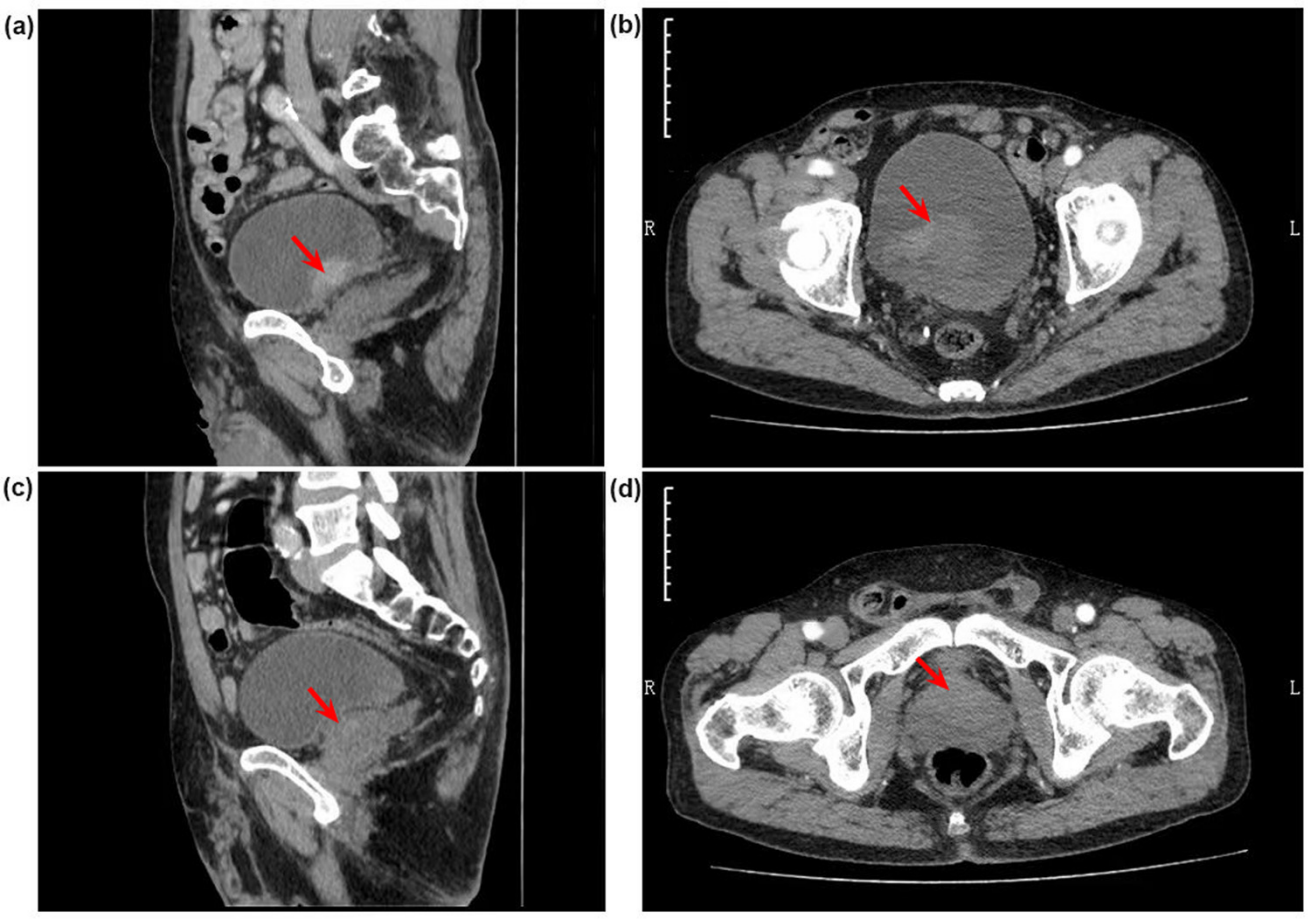
CT scan of the pelvic cavity in August 2020. (a and b) Show the bladder carcinoma: a soft tissue mass (about 38 mm × 23 mm) with a wide base was seen in the bladder triangle, and the mass was uniformly enhanced in contrast CT. The bladder wall shown with limited thickening and stiffness. (c and d) Show the prostate carcinoma: The prostate was enlarged (about 52 mm × 43 mm) with uniform density. (a and c) belongs to the sagittal position. (b and d) belongs to the axial position.

**Figure 2:**
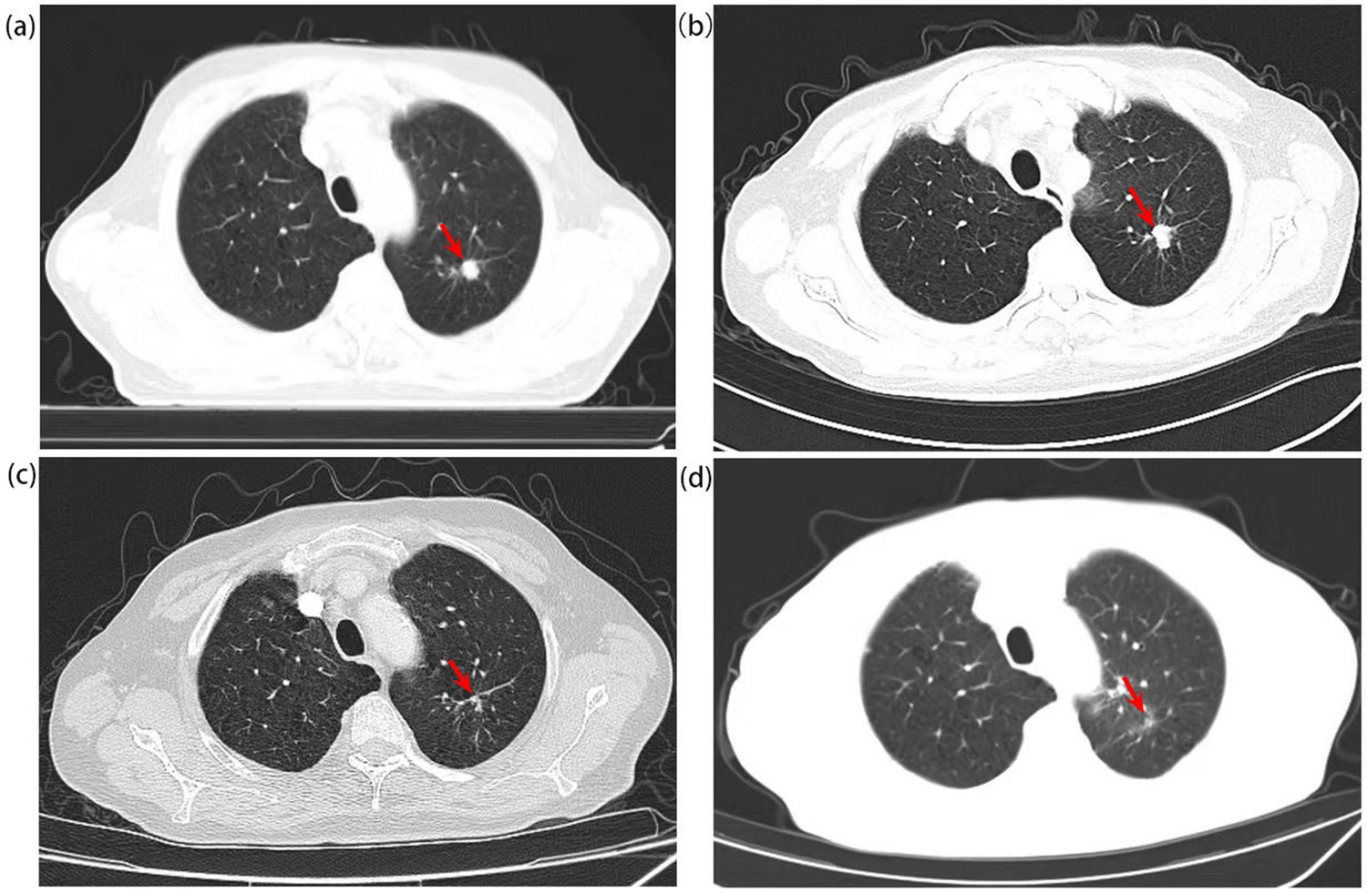
The CT scan of the chest shows the lung carcinoma: (a) shows a nodular hyperdense shadow seen in the posterior segment of the left upper lobe of the lung with medium contrast in August 2020 (with a size about 11 mm × 12 mm). (b) Shows the lesion before chemotherapy plus immunotherapy against PD-1. (c) Shows that the lesion was significantly reduced after two cycles of chemotherapy plus immunotherapy against PD-1. (d) Shows that the lesion almost disappeared after radiotherapy.

In August 2020, a 64-year-old male presented with haematuria and dysuresia for 2 weeks, both computerized tomography (CT) and ultrasound of the abdomen showed a tumour in the bladder triangle area (38 mm × 23 mm, Figure 1a and b) and another one in the prostate (Figure 1c and d). Chest CT found a nodule located in the left lung (11 mm × 12 mm, Figure 2a). The serum PSA was elevated (total PSA was 11.3 μg/L, free PSA was 1.01 μg/L). The patient had a history of smoking for 40 years, about 2 packs/day; drinking for 30 years, 150 g/day, and had quit smoking and drinking for 1 year, since he was diagnosed with hypertension 1 year ago. Besides, he underwent lower limb vein exfoliation surgery 10 years ago and repaired his left groin hernia 5 years ago.

**Figure 3:**
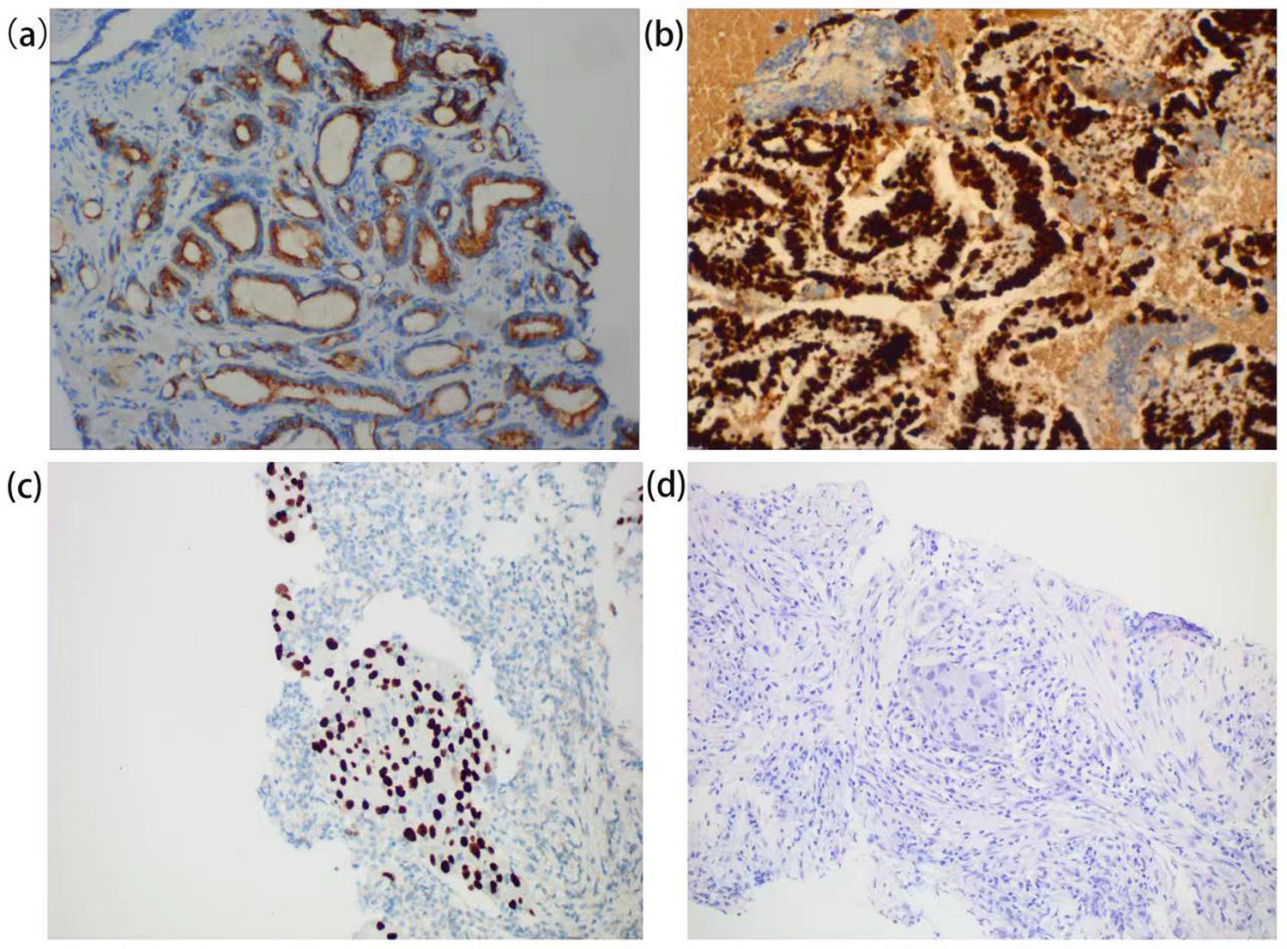
Histopathological section of the three carcinomas. (a) Shows P504S (+) in prostate adenocarcinoma. (b) Shows TTF-1 (+) in bladder urothelial carcinoma. (c and d) Show GATA-3 (+) and HE staining in NSCLC, respectively.

The prostate puncture results on August 31, 2020 indicated prostate adenocarcinoma (Figure 3a), Gleason score was 3 + 4 = 7, immunohistochemistry was PSA (−), P504S (+), P63 (−), HCK (−), ki-67 (+, about 10%), Uroplakin Ⅱ (UPK2) (−), and GATA-3 (−). The bladder cancer was partly resected on the same day, in order to release the obvious obstruction, and the pathology was bladder urothelial carcinoma (Figure 3b). Immunohistochemistry was GATA-3 (+), CK7 (+), CK20 (+), UPK (partly +), P53 (+), MLH1 (+), PMS2 (+), MSH2 (+), MSH6 (+), ki-67 (+, about 30%), and CDX2 (−).

Right after the surgery, one cycle of bladder intravesical chemotherapy (Gemcitabine, 2 g) was given immediately in the Department of Urology. The lung puncture results on September 8, 2020 was adenocarcinoma of the left lung (Figure 3c and d), immunohistochemistry showed TTF-1 (+), P40 (−), PSA (−), GATA-3 (−), CK (+), P504s (−), PD-L1 (+, TPS = 90%, CPS = 90), and microsatellite stable (MSS). The genetic testing of lung cancer tissue showed mutations in MAP2K1 (MEK1, abundance 8.46%) and TP53 (richness 4.01%), with no mutation of the driver gene, which was determined in another hospital. F-18 FDG PET/CT did not find other abnormal intake except the above three lesions.

Later, the patient underwent radical cystoprostatectomy through laparoscopy and cutaneous terminal ureterostomy in the Department of Urology on January 4, 2021. Postoperative pathological results were consistent with the previous ones: namely, prostate adenocarcinoma, Gleason score 4 + 3 = 7, immunohistochemistry showed AMACR (+), PSMA (+), HCK (−), and P63 (−); bladder urothelial carcinoma (high level), immunohistochemistry was: PCK (+), GATA-3 (+), P63 (+), CK7 (+), CK5/6 (strong +), CD44 (partial weak +), and CK20 (stove +). After the radical surgery, regular endocrine therapy began every 3 months and PSA was kept below the normal value. But soon, venous thrombosis (incomplete embolism) was found in his shallow vein of the lower limbs, and anticoagulation therapy with low molecular heparin was given. As he refused surgery and radiotherapy for the lung lesion at that time, from March 20, 2021 to June 6, 2021, he received 4 cycles of pemetrexed and carboplatin plus pembrolizumab, the evaluation was partial response (PR) after second and fourth cycles (Figure 2b and c). According to the strong requirements of the patient, bevacizumab was added in the fifth cycle on July 2, 2021. But right after that myocardial infarction occurred in July 2021 and he was in poor physical condition, so he was treated with cardiovascular diseases and all treatments against cancers were terminated. Then, from January 3, 2022 to January 14, 2022, he received radiation for his left lung lesion (50 Gy/10 fractions). After that, the lung lesion almost disappeared (Figure 2d) and he underwent regular follow up thereafter. On April 15, 2022, he received “Bentall” surgery due to the aneurysm of the aortic root. Maintenance treatment of pembrolizumab monotherapy was started and still ongoing since April 2022, and he is in good condition now and PSA is still kept below the normal value (total PSA detected in September 2022 was 0.008 μg/L, free PSA＜0.01 μg/L), no cancer recurrence or metastasis has been detected nowadays, overall survival (OS) was more than 26 months.


**Ethical statement and informed consent:** The studies involving human participants were reviewed and approved by the Medical Ethics Committee of the first affiliated hospital of North Sichuan Medical College. The patient provided his written informed consent to participate in this study, and allowed publication of this study and any accompanying images.

## 3 Discussion

Although MPMs of the prostate and bladder have been reported, NSCLC occurring with prostate and bladder cancer is rare [15,16]. In the report about synchronous triple primary malignant tumours, only a few mentioned the gene state [20]. TP53 is a well-known gene in cancer. Gene mutations such as TP53 are more likely to occur in multiple primary tumours. And MAP2K1 (MEK1) mutations were related to MAPK pathway [21,22]. In this case, tissue from NSCLC was detected with mutations in TP53 and MEK1, which suggested a poor survival. To our best knowledge, this is the first report of synchronous triple primary malignant tumours of the bladder urothelial carcinoma, prostate adenocarcinoma, and NSCLC with mutations in TP53 and MEK1, accompanied with severe cardiovascular diseases, including myocardial infarction, venous thrombosis, and aneurism of the aortic root. The severe cardiovascular diseases interrupted the patient’s treatment against cancer, but luckily, all the cancers were controlled well up to now, which demonstrates that comprehensive treatment, especially radical treatment such as operation and radiation, is very important for MPMs. In addition, patients with MEK1 mutations may have a better response to PD-1 inhibitors, which needs further research.

Smoking is a risk factor for many tumours, including lung, bladder, and kidney cancers [23]. The relationship between prostate cancer and smoking is controversial, while one meta-analysis suggests that smoking at the time of prostate cancer diagnosis is associated with an increased risk of prostate cancer-specific mortality and recurrence [24]. Smoking is associated with high risk of developing multiple primary tumours and a shorter OS [25]. Multiple pathways are involved in the process of lung carcinogenesis, and the MAPK/PI3K signalling pathway may be partially involved in the development of lung carcinogenesis in non-smoking populations [26].

For the time interval of the synchronous and metachronous tumours, the Surveillance, Epidemiology, and End Results project defines those diagnosed within 2 months to be synchronous, while the International Agency for Research on Cancer considers 6 months to be the interval [2], which is more useful. In the patient, all the prostate adenocarcinoma, bladder urothelial carcinoma, and NSCLC with mutations in TP53 and MEK1 were diagnosed almost at the same time, within 10 days, which indicated a synchronous one. According to the research, there are more metachronous tumours than synchronous ones, and synchronous ones may have a worse survival.

Besides, all the three carcinomas were treated with radical methods (operation and radiation), although the severe cardiovascular diseases interrupted his treatment against cancer, the patient was successfully treated with radical cystoprostatectomy, chemotherapy plus pembrolizumab, and radiotherapy of the lung cancer, OS was more than 26 months and maintenance treatment of pembrolizumab monotherapy was still ongoing, and he is still in good condition now and no recurrence or metastasis has been detected. This indicates that comprehensive treatment, especially radical treatment such as operation and radiation, is very important for MPMs.

## 4 Conclusion

In conclusion, we reported a synchronous triple primary malignancies of the prostate adenocarcinoma, bladder urothelial carcinoma, and NSCLC with mutations in TP53 and MEK1, though interrupted by severe cardiovascular diseases, he was successfully treated with radical cystoprostatectomy, chemotherapy plus pembrolizumab, and radiotherapy, and maintenance therapy of pembrolizumab monotherapy was still ongoing. OS was more than 26 months, which indicated that comprehensive treatment, especially radical ones, is very important for MPMs.
